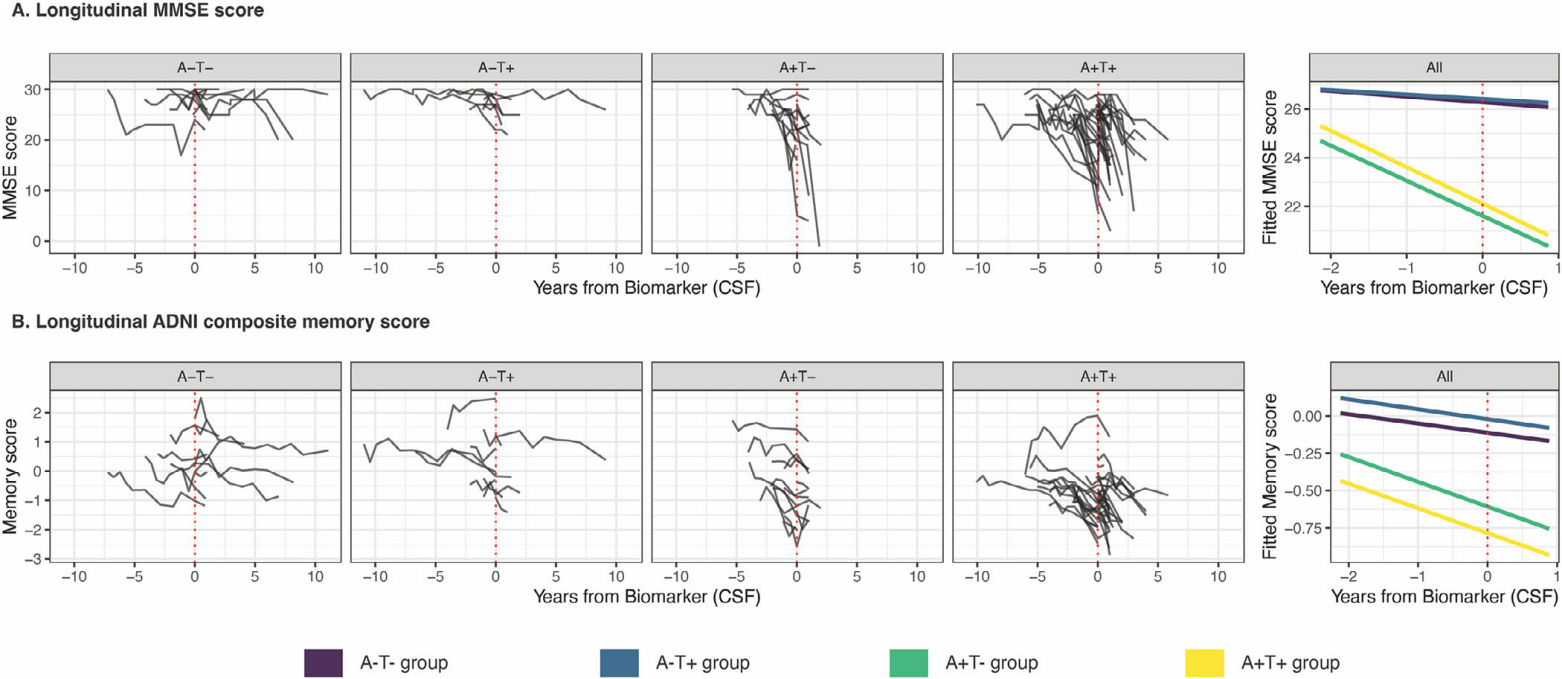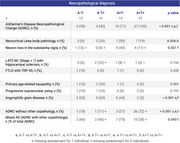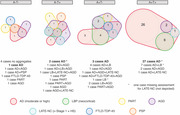# Isolated Amyloid‐β biomarker positivity: Alzheimer's disease in a mixed pathologies context?

**DOI:** 10.1002/alz.091213

**Published:** 2025-01-09

**Authors:** Konstantinos Ioannou, Khadidzha Abdullaieva, Marina Bluma, Dorota Religa, Elena Rodriguez‐Vieitez, Konstantinos Chiotis, Alzheimer's Disease Neuroimaging Initiative

**Affiliations:** ^1^ Department of Neurobiology, Care Sciences and Society, Center for Alzheimer Research, Karolinska Institutet, Stockholm Sweden; ^2^ Division of Clinical Geriatrics, Center for Alzheimer Research, Department of Neurobiology, Care Sciences and Society, Karolinska Institutet, Stockholm Sweden; ^3^ Theme Inflammation and Aging, Karolinska University Hospital, Stockholm Sweden; ^4^ Division of Neurogeriatrics, Center for Alzheimer Research, Department of Neurobiology, Care Sciences and Society, Karolinska Institutet, Stockholm Sweden; ^5^ Department of Neurology, Karolinska University Hospital, Stockholm Sweden

## Abstract

**Background:**

The in vivo amyloid‐β (A) and tau (T) biomarkers have been validated against the respective neuropathological burden of amyloid‐β plaques and neurofibrillary tangles. We aimed to assess the impact of mixed pathologies on the interpretation of AT biomarker system.

**Method:**

A subset of 71 ADNI participants with available neuropathological data and ante‐mortem cerebrospinal fluid (CSF) sampling was analyzed. The median delay between CSF sampling and death was 3.12 years (interquartile range 1.44‐5.11). AT status was determined based on CSF positivity for Aβ42 (A+; ≤981 pg/mL) and p‐tau181 (T+; >24.3 pg/mL). Linear mixed‐effects models were employed to assess the interaction of AT status with longitudinal cognitive scores. A medically trained investigator evaluated the medical history and medication use records. The neuropathological burden was assessed by the ADNI Neuropathology Core according to standard procedures. Chi‐squared statistics (α=0.05) were used to compare the frequency of clinical and neuropathological diagnoses across AT profile groups.

**Result:**

Four AT profile groups were identified: n=37 A+T+, 14 A+T‐, 10 A‐T+, and 10 A‐T‐. Both A+ groups exhibited significantly lower cognitive scores and declined significantly faster in episodic memory and executive domains over time than A‐ groups. The frequencies of psychiatric/neurological diagnoses and cerebrovascular disease risk factors were similar across groups. A+T+ and A+T‐ individuals were significantly more likely to be prescribed Alzheimer’s disease medications than A‐ individuals. The frequency of Alzheimer’s disease neuropathological change (ADNC) declined in a stepwise fashion across AT groups (A+T+ 100%; A+T‐ 71%; A‐T+ 40%; A‐T‐ 30%), and significant differences were detected between A+ and A‐ groups and within the A+ subgroups. In case of ADNC evidence, A+T‐ compared to A+T+ individuals exhibited significantly higher frequency of mixed pathologies (70% vs. 28%). Neocortical Lewy body pathology, extensive substantia nigra neurodegeneration, and argyrophilic grain disease were significantly more frequent in the A+T‐ than A+T+ individuals.

**Conclusion:**

Despite indistinguishable clinical phenotypes, A+T‐ and A+T+ groups showed distinct differences at autopsy, with higher mixed pathology burden in the A+T‐ group. Cognitive decline in A+T‐ individuals may be associated with the additional impact of mixed pathologies rather than being exclusively linked to AD neuropathology.